# Highly expressed CENPL is correlated with breast cancer cell proliferation and immune infiltration

**DOI:** 10.3389/fonc.2023.1046774

**Published:** 2023-02-02

**Authors:** Zhengwei Gui, Yao Tian, Shiyang Liu, Tianyao Yu, Chenguang Liu, Lin Zhang

**Affiliations:** Department of Thyroid and Breast Surgery, Tongji Hospital, Tongji Medical College, Huazhong University of Science and Technology, Wuhan, Hubei, China

**Keywords:** CENPL, breast cancer, cell proliferation, immune infiltration, oncogene

## Abstract

**Background:**

Centromere protein L (CENPL) is associated with a variety of human diseases. However, its function in breast cancer remains uncertain.

**Methods:**

The Cancer Genome Atlas (TCGA) and genotype-tissue expression across cancer data were used to investigate CENPL expression. Using TCGA clinical survival data, the relationship between CENPL expression and patient prognosis was assessed. Using the cluster profiler R software tool, enrichment analysis of CENPL was carried out. Additionally, by studying the TCGA database, the relationship between CENPL expression and immune cell infiltration was assessed. To evaluate CENPL’s impact on breast cancer cell proliferation, the CCK8 test and colony-formation assay were carried out. Scratch testing and the transwell assay were used to evaluate the effects of CENPL on breast cancer cell migration.

**Results:**

Breast cancer was one of numerous tumor forms with high CENPL expression. Significant relationships between high CENPL expression and the cell cycle, nuclear division, organelle fission, and chromosome segregation were found. Further investigation revealed that minimal infiltration of CD8-positive T cells and natural killer (NK) cells and high levels of Tregs and macrophages were correlated with high levels of CENPL expression. CENPL expression was linked to more than half of the ICP genes. Breast cancer cells’ ability to proliferate and migrate was decreased by CENPL knockdown.

**Conclusions:**

Our findings suggest that CENPL may be an oncogene in breast cancer and a predictor of efficacy of immunotherapy for breast cancer.

## Introduction

Breast cancer is the most frequently diagnosed cancer and the second most common cause of cancer mortality in women worldwide (1). Approximately 10% of cases are caused by changes in inherited genes, like those in BRCA1 and BRCA2. Due to tumor heterogeneity and drug resistance, multimodality treatment includes chemotherapy, endocrine therapy, and targeted therapies—reduces the effectiveness of treatment. For the multimodality treatment of breast cancer, it is essential to discover new driver genes and biomarkers. Thanks to the rapid development of transcriptomics and high-throughput sequencing technology, more and more driver genes are being identified. The subsequent stages of clinical diagnosis and treatment can be aided by identifying and verifying the essential genes of them. The centromere protein (CENP) family includes centromere protein L (CENPL), which is required for normal cell division (mitosis and meiosis). CENPL is crucial for recruiting other centromere components to come together to form centromeres and can be combined with CENPN to locate CENPA nucleosomes. CENPL may act as a possible biomarker and oncogene in LUAD, according to recent investigations (2). Elevated CENPL may be a potential prognostic marker and connected with immune infiltration in HCC (3). However, the relationship of CENPL with breast cancer remains unknown.

In this study, we examined the relationship between CENPL expression and clinicopathological characteristics and prognosis in diverse cancers from The Cancer Genome Atlas (TCGA). We also investigated at just how CENPL expression correlates to breast cancer’s molecular pathways. We also examined the link between CENPL expression and immune cell infiltration because it is essential for patients with breast cancer to have immune cell infiltration. Finally, we assessed at how CENPL knockdown affected breast cancer cells’ ability to proliferate and migrate. Our findings imply that CENPL might be a viable therapeutic target and biomarker.

## Materials and methods

### Data collection and processing

We obtained the mRNA expression profiles and clinical information of breast cancer patients from the Genotype-Tissue Expression Project (GTEx) database (n = 179) and the Cancer Genome Atlas (TCGA) database (n = 1065). Transcripts per million reads were used to standardize the level 3 HTSeq-FPKM format data (TPM). For pan-cancer analysis, we additionally got the RNA-sequencing data in TPM format from the UCSC Xena database and the GTEx database. Using a web-based database called Kaplan-Meier Plotter (www.kmplot.com) (4), that contains data on gene expression and patient survival rates for BC patients, the predictive significance of CENPL mRNA expression was investigated. To onfirm the protein level, we additionally gathered immunohistochemistry (IHC) images from the Human Protein Atlas (HPA) database (http://www.proteinatlas.org/) (5, 6).

### Correlation and enrichment analyses

A Pearson correlation analysis of the mRNAs CENPL and other mRNAs in breast cancer was performed using TCGA BRCA data. The 300 genes that had the strongest positive correlation with CENPL were chosen for enrichment analysis in order to determine the function of CENPL. Using the EnrichGO function in the clusterProfile R software package, gene ontology (GO) analysis was done.

### Cell culture and treatment

Breast cancer cell lines MCF7, MDA-MB-231, MDA-MB-468, and SKBR3 were purchased from the Shanghai Institute of Cell Biology (Shanghai, China). MCF7, MDA-MB-231, and SKBR3 cells were grown in Dulbecco’s Modified Eagle Medium (DMEM) plus 10% FBS. MDA-MB-468 cells were grown in RPMI 1640 plus 10% FBS. All cells were cultured in a 37 °C humidified incubator with 5% CO2. All cancer cell lines were authenticated using STR profiling

### RNA extraction and qRT-PCR

Total RNA from approximately 1×106 cells was isolated using TRIzol reagent (Invitrogen, US) according to the manufacturer’s protocol. The primers used for qRT-PCR, including those for CENPL and GAPDH, were obtained from Tsingke (Tsingke, China). The primer sequences were (5’-3’): CENPL forward-CACCAGAGTCAACTCCTAGTG, reverse- TCTGCTTCCTGACCGATTCTAA. GAPDH forward- GGAGCGAGATCCCTCCAAAAT. reverse- GGCTGTTGTCATACTTCTCATGG. QRT-PCR parameters were: 95°C 5 min; (95°C 5 s, 60°C 30 s) × 40 amplification cycles. Relative expression levels were normalized to internal controls and calculated according to the 2–ΔΔCT method.

### CCK8 assay

Cells in the logarithmic growth phase from each experimental group were trypsinized, and resuspended in a complete medium. According to the manufacturer’s protocol, we assessed the cell proliferation by Cell Counting Kit-8 (Invitrogen, US) assay on day1 (24 hours after inoculation), day2 (48 hours after inoculation), day3 (72 hours after inoculation), day4 (96 hours after inoculation). OD values at 450 nm were recorded by a microplate reader(Molecular Devices, Rockford, IL, USA).

### Colony-formation assay

Two thousand breast cancer cells were plated into six-well plates and cultured for 7 days. Cell colonies were fixed with 4% formaldehyde for 15 min and stained with 0.5% crystal violet, and then imaged.

### Transwell assay

Thirty thousand breast cancer cells were plated in the upper chamber of the transwell chamber (Corning, USA) in a 24-well plate. 24 h after incubation at 37°C, cells on the top surface of the chamber were removed by wiping. Cells on the bottom surface of the chamber were fixed with 4% paraformaldehyde for 15 min and stained with crystal violet (Beyotime, China) for 5 min. The number of migrated cells was imaged and counted.

### Scratch test

We utilized a scratch assay to measure cell migration. Two-well culture inserts (Ibidi, Germany) were inserted into 24-well cell culture plate wells. MDA-MB-231 and MCF-7 cells in the logarithmic growth phase were inoculated in culture-inserts wells. The cells were cultured for 24h in an incubator. The insert was carefully removed with sterile forceps and 1 mL serum-free media was added per well. The migratory and invasive cells were imaged with a light microscope under a high magnification objective at 0 hours and 24 hours after removing the culture-inserts.

### Immune cell infiltration

The immune cell infiltration in breast cancer was analyzed using the GSVA package (version 1.34.0) (7) in R (version 3.6.3). The ssGSEA test served as the basis for the results. Based on earlier research, immune cell classification and marker establishment were carried out (8). According to the median CENPL expression (high versus low level), TCGA breast cancer samples were split into two groups, and their immune cell infiltration levels were contrasted.

### Statistical analyses

Standard deviation and means are used to express data. Software SPSS version 26.0 was used to examine each piece of data (SPSS, Inc., Chicago, IL, USA). The Kolmogorov-Smirnov test was used to determine whether the data were normal. The Student’s t-test was used to evaluate pairwise differences between groups. The cutoff for significance was P <0.05.

## Results

### Patient characteristics

The 1,065 breast cancer patients in our cohort had clinical data and RNA-sequencing results, and 113 of them had matched samples of nearby normal tissue that were obtained from TCGA. Additionally, we retrieved the gene expression data of normal breast tissues (n = 179) from the GTEx database to enhance the sample size of normal breast tissues.

### CENPL expression analysis

**Figure 1:**
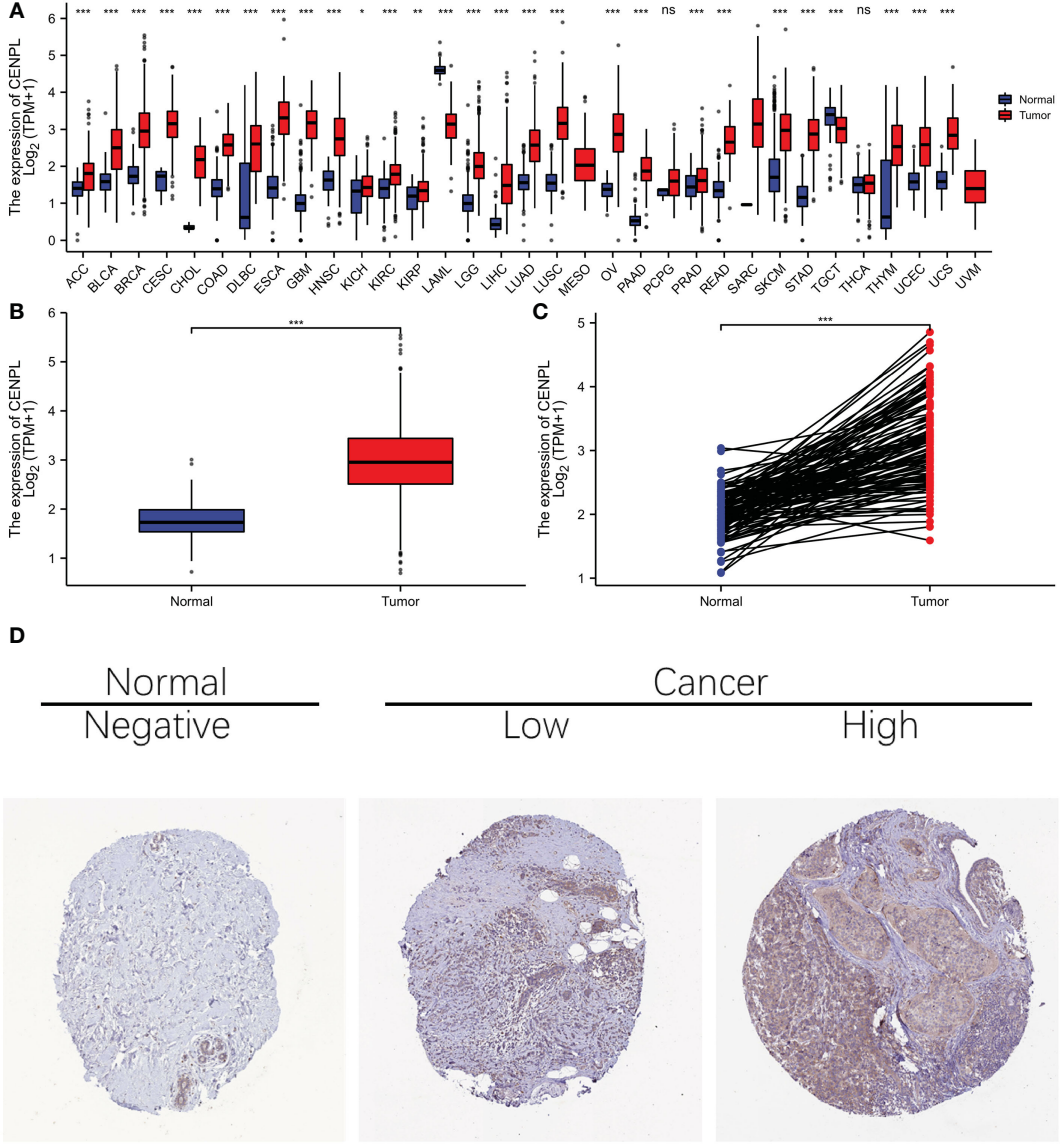
The expression difference of CENPL in cancer tissue and normal tissue. **(A)** Expression of CENPL in pan-cancer and adjacent normal tissues in TCGA and GTEx databases. **(B)** expression of CENPL in unpaired breast cancer samples in TCGA-Brca database. **(C)** expression of CENPL in paired breast cancer samples in TCGA-Brca database. **(D)** immunohistochemistry (IHC) images from the Human Protein Atlas (HPA) database Data were shown as mean ± SD. *p < 0.05, **p < 0.01, ***p < 0.001.

As we first investigated at CENPL expression in the TCGA and GTEx pan-cancer databases, we discovered that 30 cancers had higher CENPL expression than the comparable normal tissues, including ACC、 BLCA、 BRCA、 CESC、 CHOL、 COAD、 DLBC、 ESCA、 GBM、 HNSC、 KICH、 KIRC、 KIRP、 LAML、 LGG、 LIHC、 LUAD、 LUSC、 MESO、 OV、 PAAD、 PRAD、 READ、 SARC、 SCKM、 STAD、 TGCT、 THYM、 UCEC AND UCS (**Figure 1A**).

With regard to BRCA breast cancer in the TCGA cohort, elevated CENPL expression was seen in both the unpaired samples (**Figure 1B**) and the paired samples (**Figure 1C**), and images of protein expression of CENPL were obtained from the HPA (**Figure 1D**), suggesting that CENPL may play a role in the pathogenesis of breast cancer.

### Association between CENPL expression and breast cancer patient prognosis

**Figure 2:**
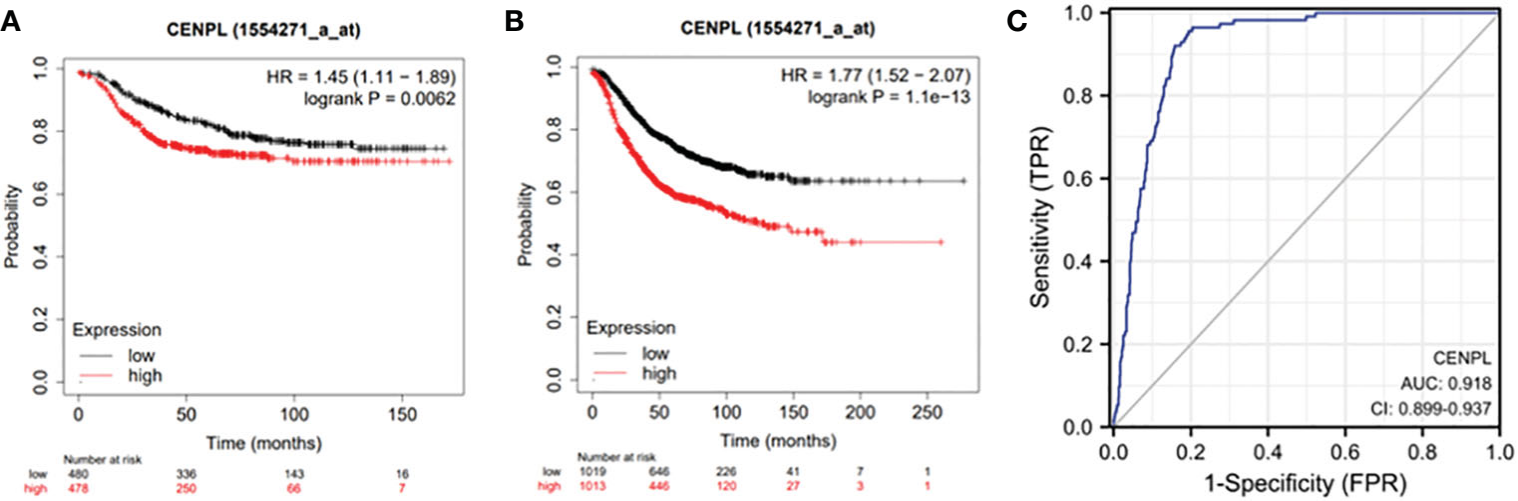
Expression of CENPL and prognosis of breast cancer patients. **(A)** RFS of breast cancer patients based on CENPL expression level and **(B)** DMFS of breast cancer patients based on CENPL expression level.

We looked into the connection between CENPL expression and distant metastasis-free survival (DMFS) and relapse-free survival (RFS) in the TCGA cohort to analyze the efficacy of CENPL expression in predicting the prognosis of breast cancer patients. (**Figure 2**). The findings demonstrated that patients had a worse prognosis when CENPL expression is high.(RFS P=1.1e-13,DMFS P=0.0062)

### Associations between CENPL expression and clinicopathologic variables

**Figure 3:**
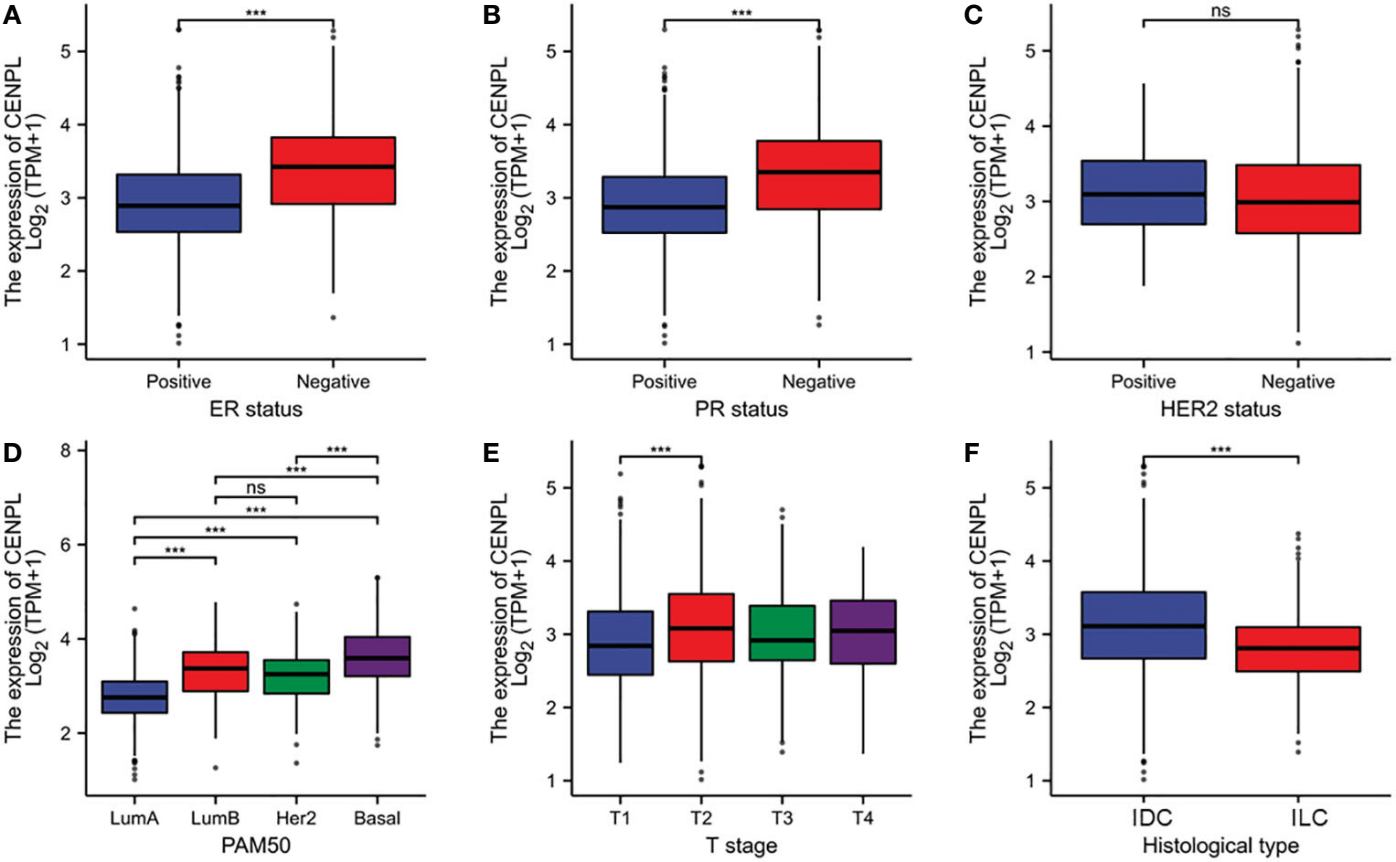
Relationship between CENPL expression and clinicopathologic features of breast cancer patients. **(A)** ER status based on CENPL expression level. **(B)** PR status based on CENPL expression level. **(C)** Her-2 status based on CENPL expression level. **(D)** Pathological type based on CENPL expression level. **(E)** T stage based on CENPL expression level. **(F)** histological type based on CENPL expression level.

As shown in **Table S1** and **Figure 3**, high expression of CENPL was significantly associated with T stage (T1 vs. T2, p = 0.003), ER status (p < 0.001), PR status (p < 0.001), histological type (p < 0.001), PAM50 (Luminal B vs. Luminal A, p < 0.001; human epidermal growth factor receptor 2 [HER2] vs. Basal, p < 0.001; Luminal vs. Basal, p< 0.001)‵

### Correlation and enrichment analyses

**Figure 4:**
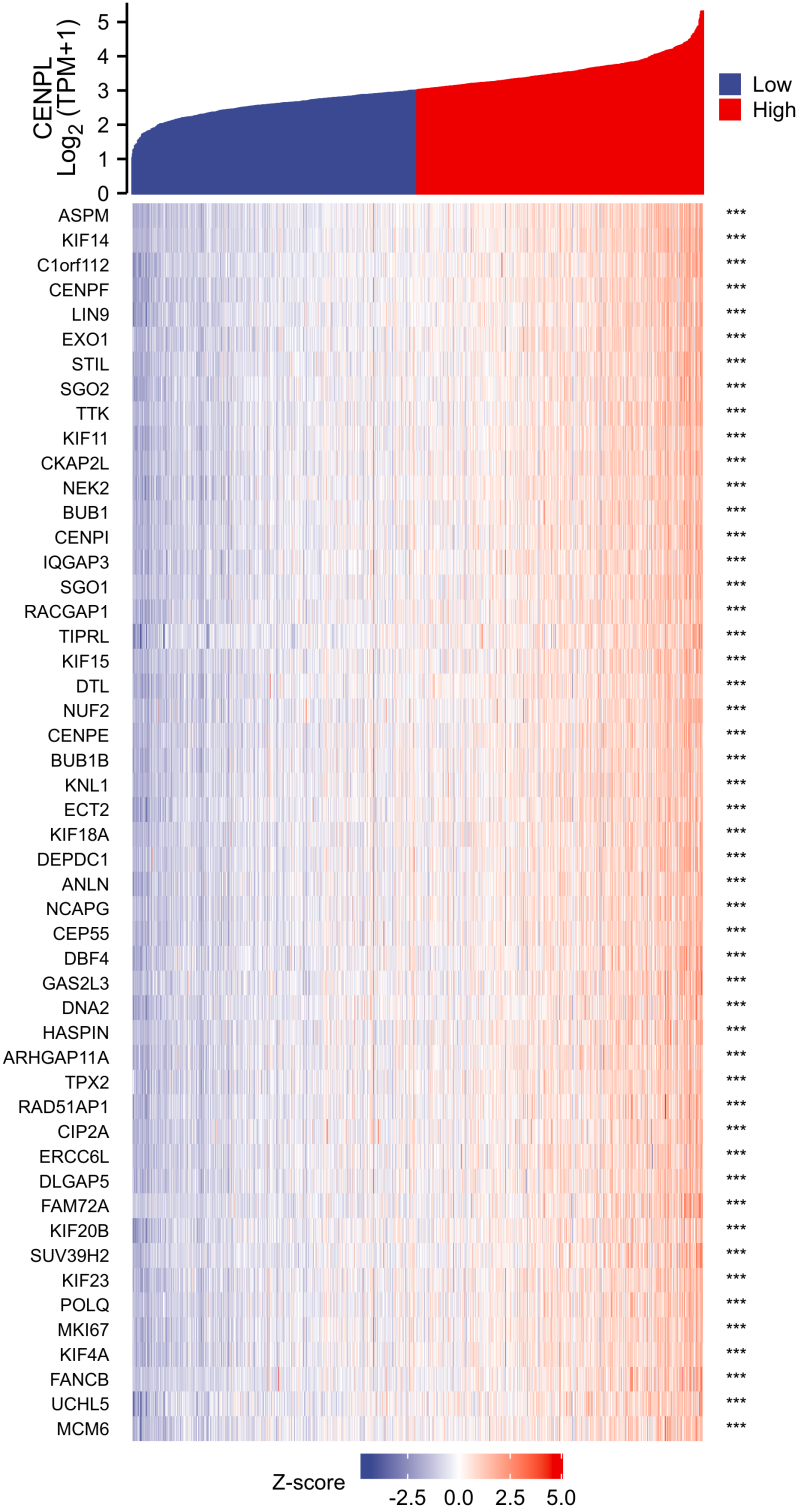
The 50 co-expressed genes with the highest positive correlation of CENPL.

**Figure 5:**
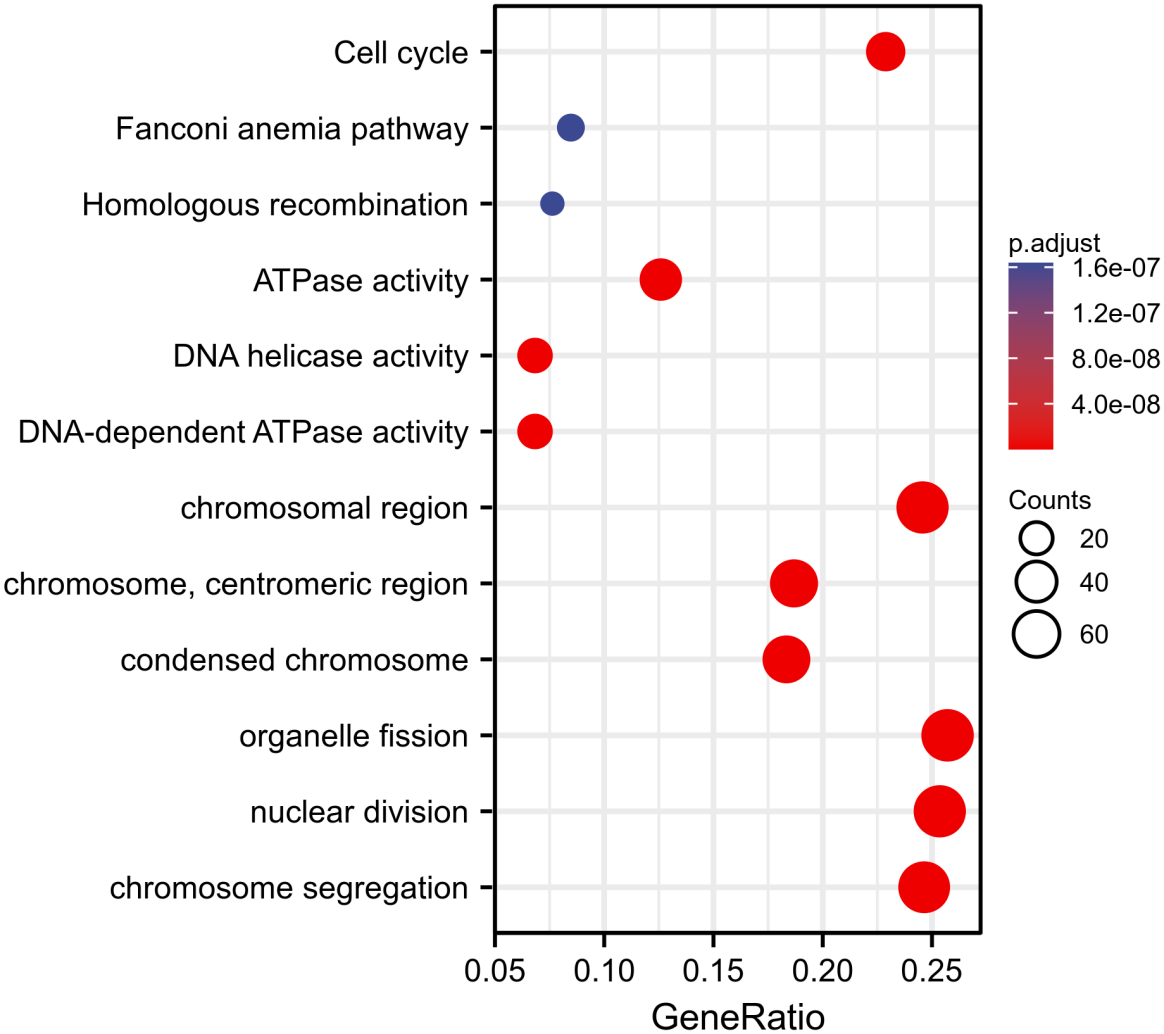
Pathway enrichment analysis of CENPL.

We used TCGA data to conduct correlation analysis between CENPL and all other mRNAs in breast cancer in order to more effectively investigate the roles and pathways involved by CENPL. The top 50 genes were displayed as a heatmap using the 300 genes that were picked for enrichment analysis and were most strongly related with CENPL (**Figure 4**). Using the clusterProfiler tool of the R programming language, we further investigated putative functional pathways based on the top 300 genes. CENPL was mostly linked to the cell cycle, organelle fission, nuclear division, and chromosome segregation, according to GO functional enrichment analysis (**Figures 5**). The Kyoto Encyclopedia of Genes and Genomes (KEGG) databases were searched using gene set enrichment analysis. In light of these findings, breast cancers with high CENPL expression have multiple oncogenic pathways hyperactivated, including those that regulate cell proliferation.

### Correlation between immune cell infiltration and CENPL expression

**Figure 6:**
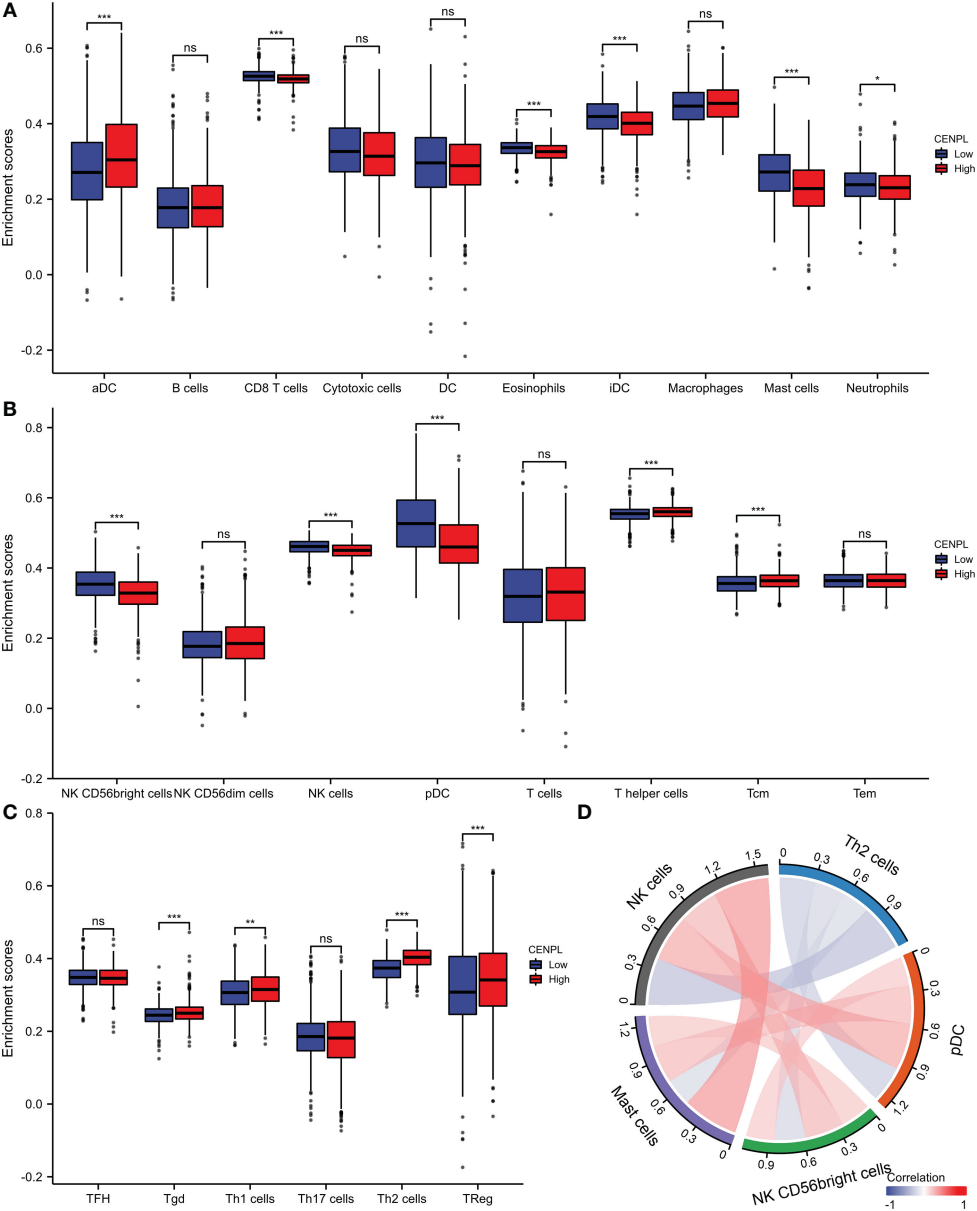
Analysis of CENPL and immune cell infiltration groups. **(A-C)** Grouping of immune cells based on CENPL expression levels. **(D)** Correlation of 5 immune cells.

**Figure 7:**
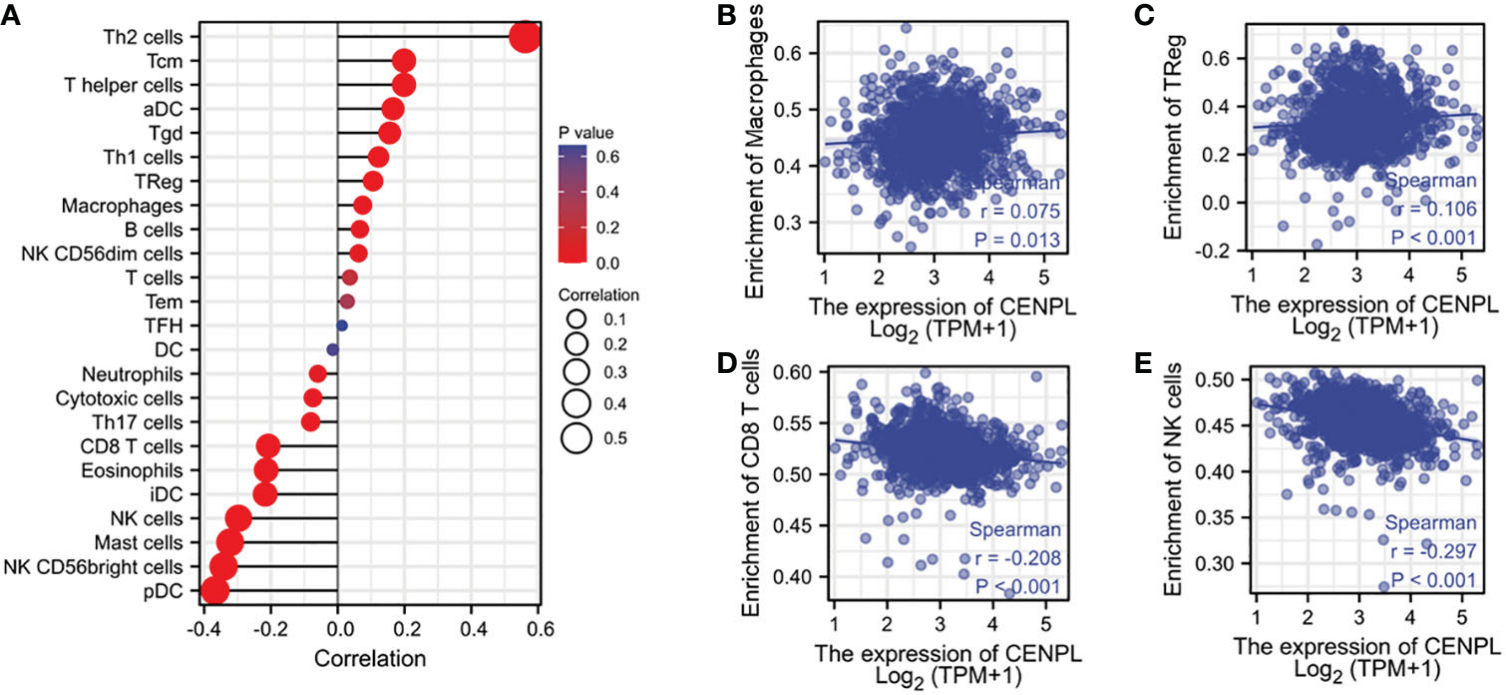
Associated between CENPL with immune cell infiltration. **(A)** Correlation between the expression level of CENPL and various immune cell infiltration. **(B)** Correlation between CENPL expression and macrophages. **(C)** Correlation between CENPL expression and Tregs. **(D)** Correlation between CENPL expression and CD8+T cells. **(E)** correlation between CENPL expression and NK cells.

The TCGA database was then used to estimate the immune cell infiltration of breast cancer patients. High CENPL expression promotes the intratumoral accumulation of macrophage infiltration and Tregs cell infiltration, as demonstrated by the significant positive association between CENPL expression and macrophage infiltration and Tregs cell infiltration and the negative correlation between CENPL expression and CD8+ T cells and NK cells (**Figures 6**, **7**). These findings imply a strong correlation between high CENPL expression and breast cancers that are immune-activated.

### CENPL knockdown inhibited the malignant behaviors in breast cancer cells

**Figure 8:**
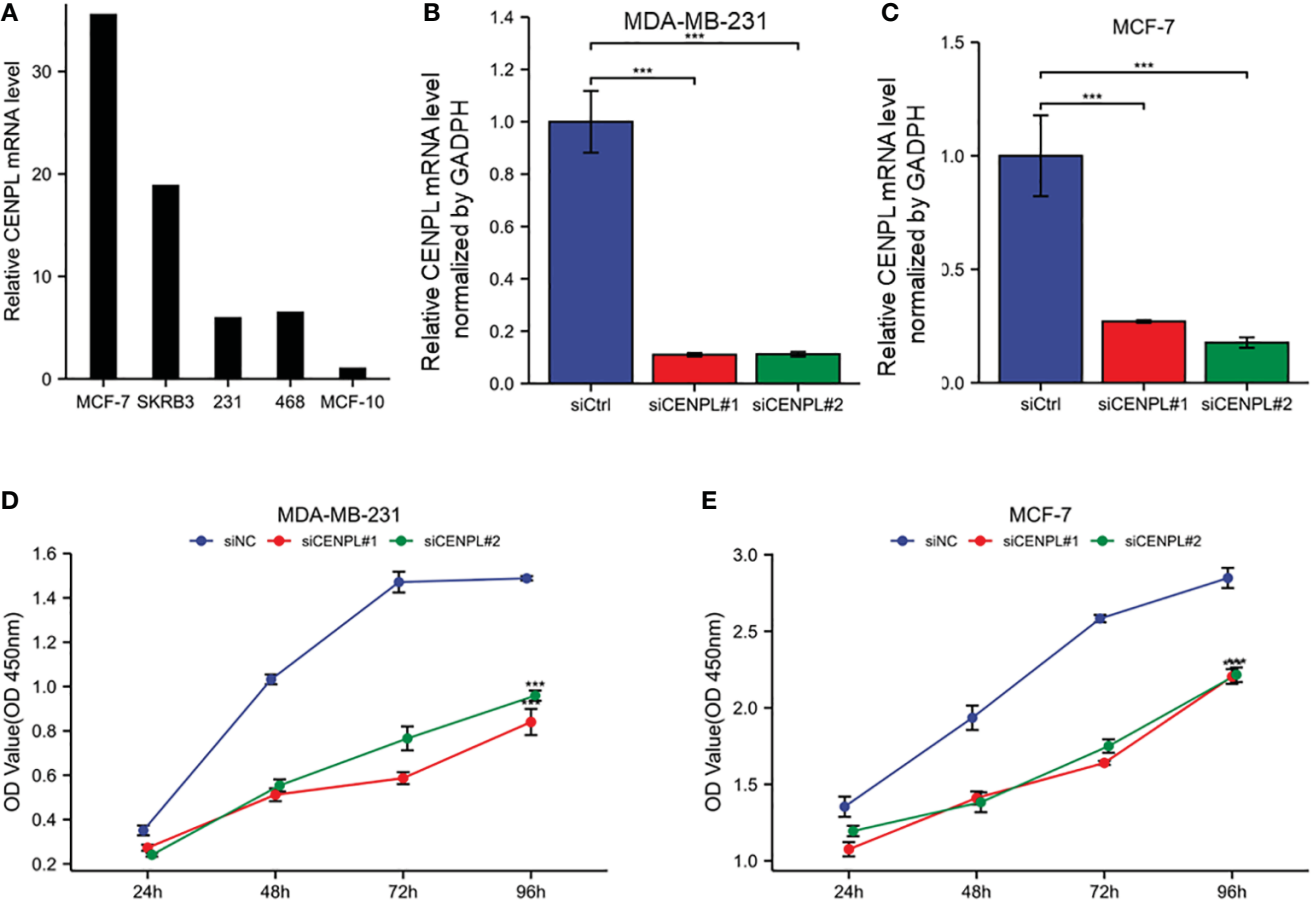
Expression and knockdown of CENPL in various cell lines and CCK8 cell proliferation experiment. **(A)** CENPL expression in MCF7, SKBRE3, MDA-MB-231, MDA-MB-468 and other breast cancer cell lines and MCF10A cell lines. **(B)** CENPL knockdown efficiency of two SiRnas in MDA-MB-231 cell lines. **(C)** knockdown of two SiRnas in MCF7 cell lines Efficiency of CENPL. **(D-E)** Cell proliferation in two siRNA knockout groups and control groups in MDA-MB-231 and MCF7 cell lines.

**Figure 9:**
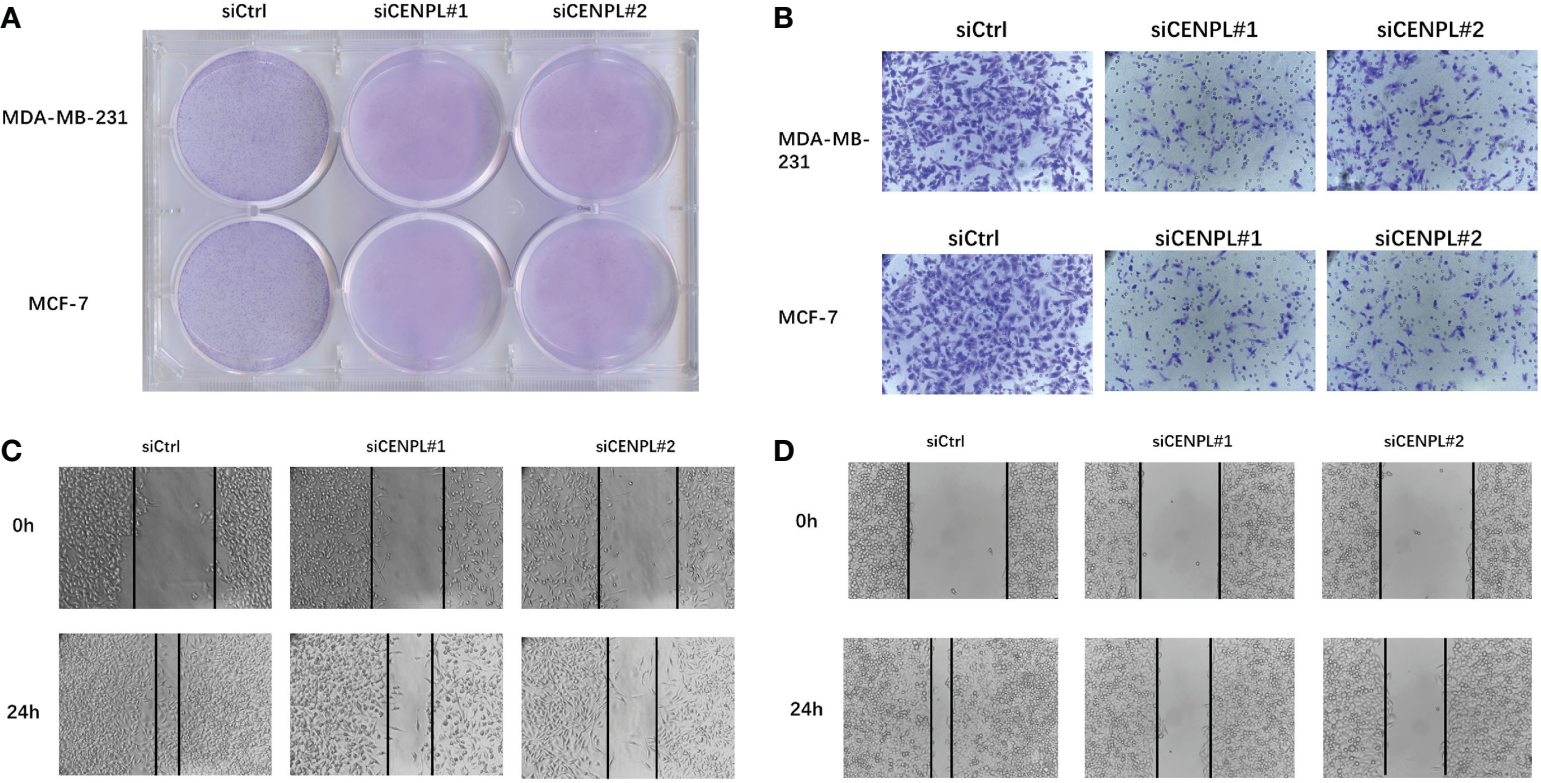
Clone formation experiment, Transwell experiment and scratch experiment. **(A)** Clone formation of control group and two siRNA knockout groups in MDA-MB-231 and MCF7 cell lines. **(B)** Transwell images of control group and two siRNA knockout groups in MDA-MB-231 and MCF7 cell lines. **(C–D)** Scratch test images of control group and two siRNA knockout groups in MDA-MB-231 and MCF7 cell lines.

Next, we evaluated CENPL expression in multiple breast cancer cell lines and found relatively higher expression of CENPL in MCF-7, MDA-MB-231, MDA-MB-468, and SKBR3 cells compared with that in MCF-10 cells (**Figure 8A**). To explore the biological effects of CENPL on breast cancer cell proliferation, we knocked down CENPL expression in MDA-MB-231 (**Figure 8B**) and MCF-7 (**Figure 8C**) cells *via* two CENPL siRNA, and validated the successful silence of CENPL expression in these two cell lines. Next, we conducted a CCK8 assay to evaluate cell proliferation. The Results showed that the proliferation rates of the MDA-MB-231 and MCF-7 cells were significantly inhibited following CENPL knockdown (**Figures 8D–E**). The negative effect of CENPL on breast cancer cell proliferation was further confirmed by colony-formation assay (**Figure 9A**). Besides, CENPL silencing also significantly impaired the migratory ability of breast cancer cells as evidenced by the Transwell assay (**Figure 9B**) and scratch test (**Figures 9C, D**). Taken together, CENPL facilitates the proliferation and migration of breast cancer cells.

### CENPL expression is related to immune checkpoint (ICP) genes in breast cancer

**Figure 10:**
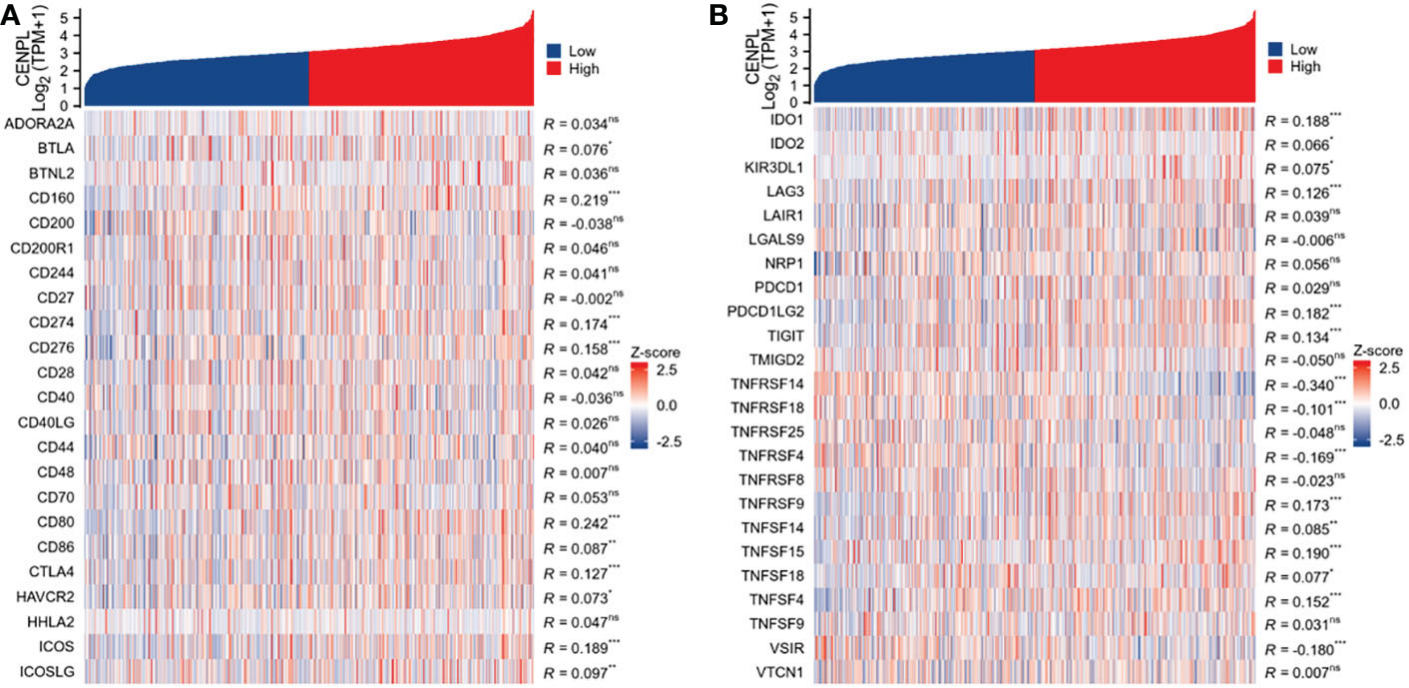
Co-expression of CENPL and immune checkpoint related genes.

Immune checkpoint genes have a substantial effect on immune cell infiltration and immunotherapy, according to studies (9). We chose 47 genes that have previously been linked to immunological checkpoints in studies and investigated the co-expression of CENPL with the above 47 genes, as shown in **Figure 10** to examine the relationship between immune checkpoints and CENPL. Among them, 25 genes including BTLA, CD160, CD274, CD276, CD80, CD86, CTLA4, HAVCR2, ICOS, ICOSLG, IDO1, IDO2, KIR3DL1, LAG3, PDCD1LG2, TIGIT, TNFRSF14, TNFRSF18, TNFRSF4, TNFRSF9, TNFSF14, TNFSF15, TNFSF18, TNFSF4, VSIR were associated with CENPL expression, among which only four genes including TNFRSF14, TNFRSF18, TNFRSF4, VSIR were negatively correlated with CENPL expression, while the remaining 21 genes were positively correlated with CENPL expression.

## Discussion

Breast cancer is the most often diagnosed cancer in women, representing for 25% of all female cancer cases worldwide and a higher percentage among young women. Due to tumor heterogeneity and drug resistance, the current treatment for breast cancer, which consists of surgery, radiation, chemotherapy, hormone therapy, and targeted therapy, has a lot of space for improvement. There is currently no effective conventional treatment that is approved for triple-negative breast cancer (10). Finding and confirming the essential driver genes may offer fresh perspectives on breast cancer treatment options.

CENPs overexpression has been reported in many cancers (11–13). Recent studies have shown that CENPL may function as a potential biomarker and oncogene in LUAD and HCC.

We examined the expression level of CENPL using pan-cancer TCGA and GTEx data obtained from the UCSC Xena database. Compared with normal tissues, we found that CENPL was highly expressed in ACC、 BLCA、 BRCA、 CESC、 CHOL、 COAD、 DLBC、 ESCA、 GBM、 HNSC KICH、 KIRC、 KIRP、 LAML、 LGG、 LIHC、 LUAD、 LUSC、 MESO、 OV、 PAAD、 PRAD READ、 SARC、 SCKM、 STAD、 TGCT THYM、 UCEC AND UCS, suggesting that it may act as an oncogene.

According to the findings of our enrichment analysis, CENPL is strongly linked to pathways that are involved in cell proliferation, including the cell cycle, organelle fission, nuclear division, and chromosome segregation. Cellular research verified that CENPL knockdown dramatically reduced the proliferation and migration of breast cancer cells.

People gradually come to realize that conventional TNM staging and pathological grading can only provide a limited amount of prognostic information and cannot predict the response to treatment as medicine progresses. The host’s immune system, which is critical for regulating tumor development and progression as well as predicting prognosis and therapeutic response, is becoming more and more of a concern for researchers (14, 15). The tumor microenvironment’s stroma cells, particularly immune cells, are essential components that have a significant effect on the tumor cells’ malignant behaviors (16–18). The accumulation of regulatory T cells (Tregs), tumor-associated macrophages, and other formidable lymphocytes that play important anti-tumor roles have all been found to be directly related to tumor progression in the previous (19, 20). Although many other immune cell types participate in the cancer immunity cycle, CD8 + cytotoxic T lymphocytes are primarily responsible for recognizing and eliminating tumor cells (21, 22). Effector T cells and CD8+ T cells can grow and function more slowly when treg cells are present. Macrophages can enhance tumor cell migration and invasion and promote angiogenesis (23, 24). According to our research, high levels of CENPL expression were significantly positively connected with Treg cell and macrophage infiltration and negatively correlated with CD8+ T cells and NK cells. This suggests that CENPL plays a critical role in regulating tumor immunology. CENPL expression was linked to more than half of the ICP genes, implying that CENPL may coordinate the activity of these ICP genes in different signal transduction pathways and may be a target for immunotherapy. High CENPL expression may predict better efficacy of immunocheckpoint inhibitor therapy and thus has potential as an efficacy predictor.

In conclusion, CENPL may act on immune cells that have invaded the tumor as well as tumor cells to cause a harmful role in the development of breast cancer.

Our study does, however, have certain drawbacks. Our findings imply that CENPL might be a driver gene in breast cancer; however, as its mode of action is yet unknown, more research is required.

## Data availability statement

The original contributions presented in the study are included in the article/**Supplementary Material**. Further inquiries can be directed to the corresponding author.

## Author contributions

LZ, ZG, and YT contributed to conception and design of the study. ZG organized the database. SL and TY performed the statistical analysis. ZG wrote the first draft of the manuscript. ZG and CL wrote sections of the manuscript. All authors contributed to the article and approved the submitted version.
