# Supplementary material for: Highly expressed CENPL is correlated with breast cancer cell proliferation and immune infiltration
Source: Front Oncol. 2023 Feb 2;13:1046774. doi: 10.3389/fonc.2023.1046774 (PMC9932532; doi:10.3389/fonc.2023.1046774)
Supplement: Supplementary file 1 [file Table_1.docx]

| Characteristic | Low expression of CENPL | High expression of CENPL | p |
| --- | --- | --- | --- |
| n | 532 | 533 |  |
| Age, n (%) |  |  | 0.443 |
| <=60 | 287 (26.9%) | 301 (28.3%) |  |
| >60 | 245 (23%) | 232 (21.8%) |  |
| T stage, n (%) |  |  | **0.003** |
| T1 | 160 (15.1%) | 115 (10.8%) |  |
| T2 | 278 (26.2%) | 337 (31.7%) |  |
| T3 | 75 (7.1%) | 62 (5.8%) |  |
| T4 | 17 (1.6%) | 18 (1.7%) |  |
| N stage, n (%) |  |  | 0.246 |
| N0 | 238 (22.8%) | 269 (25.7%) |  |
| N1 | 183 (17.5%) | 166 (15.9%) |  |
| N2 | 56 (5.4%) | 60 (5.7%) |  |
| N3 | 42 (4%) | 32 (3.1%) |  |
| M stage, n (%) |  |  | 0.649 |
| M0 | 424 (46.6%) | 465 (51.2%) |  |
| M1 | 8 (0.9%) | 12 (1.3%) |  |
| Pathologic stage, n (%) |  |  | 0.058 |
| Stage I | 101 (9.7%) | 79 (7.6%) |  |
| Stage II | 282 (27.1%) | 324 (31.1%) |  |
| Stage III | 129 (12.4%) | 109 (10.5%) |  |
| Stage IV | 8 (0.8%) | 10 (1%) |  |
| Race, n (%) |  |  | 0.483 |
| Asian | 27 (2.8%) | 33 (3.4%) |  |
| Black or African American | 91 (9.3%) | 88 (9%) |  |
| White | 389 (39.9%) | 348 (35.7%) |  |
| Histological type, n (%) |  |  | **< 0.001** |
| Infiltrating Ductal Carcinoma | 334 (34.8%) | 423 (44.1%) |  |
| Infiltrating Lobular Carcinoma | 135 (14.1%) | 67 (7%) |  |
| ER status, n (%) |  |  | **< 0.001** |
| Negative | 67 (6.6%) | 170 (16.7%) |  |
| Indeterminate | 0 (0%) | 2 (0.2%) |  |
| Positive | 442 (43.5%) | 336 (33%) |  |
| PR status, n (%) |  |  | **< 0.001** |
| Negative | 114 (11.2%) | 224 (22%) |  |
| Indeterminate | 1 (0.1%) | 3 (0.3%) |  |
| Positive | 394 (38.8%) | 280 (27.6%) |  |
| HER2 status, n (%) |  |  | 0.048 |
| Negative | 275 (38.4%) | 273 (38.1%) |  |
| Indeterminate | 9 (1.3%) | 3 (0.4%) |  |
| Positive | 67 (9.3%) | 90 (12.6%) |  |
| PAM50, n (%) |  |  | **< 0.001** |
| Normal | 35 (3.3%) | 5 (0.5%) |  |
| LumA | 376 (35.3%) | 175 (16.4%) |  |
| LumB | 56 (5.3%) | 146 (13.7%) |  |
| Her2 | 30 (2.8%) | 52 (4.9%) |  |
| Basal | 35 (3.3%) | 155 (14.6%) |  |
| Menopause status, n (%) |  |  | 0.555 |
| Pre | 110 (11.5%) | 114 (11.9%) |  |
| Peri | 16 (1.7%) | 23 (2.4%) |  |
| Post | 346 (36.2%) | 347 (36.3%) |  |
| Anatomic neoplasm subdivisions, n (%) |  |  | 0.071 |
| Left | 261 (24.5%) | 292 (27.4%) |  |
| Right | 271 (25.4%) | 241 (22.6%) |  |
| radiation_therapy, n (%) |  |  | 0.312 |
| No | 231 (23.8%) | 201 (20.7%) |  |
| Yes | 270 (27.8%) | 270 (27.8%) |  |
| OS event, n (%) |  |  | 0.159 |
| Alive | 467 (43.8%) | 451 (42.3%) |  |
| Dead | 65 (6.1%) | 82 (7.7%) |  |
| DSS event, n (%) |  |  | 0.310 |
| Alive | 492 (47%) | 473 (45.2%) |  |
| Dead | 36 (3.4%) | 45 (4.3%) |  |
| Age, median (IQR) | 59 (49, 68) | 58 (48, 67) | 0.390 |
